# Early-flowering sweet orange mutant ‘x11’ as a model for functional genomic studies of *Citrus*

**DOI:** 10.1186/1756-0500-7-511

**Published:** 2014-08-10

**Authors:** Thaísa Tessutti Pinheiro, Antonio Figueira, Rodrigo Rocha Latado

**Affiliations:** Centro de Energia Nuclear na Agricultura, Universidade de São Paulo, Av. Centenário, 303, CP 96, Piracicaba, SP 13400-970 Brazil; Centro de Citricultura “Sylvio Moreira”, Instituto Agronômico, CP 04, Cordeirópolis, SP 13490-970 Brazil

**Keywords:** Flower, Fruit, Gene, Juvenility, Model system, Transgenic

## Abstract

**Background:**

There had been many reports on genetic transformation of *Citrus* for functional genomic studies but few included genes associated with flower or fruit traits. A major reason for this might derive from the extensive juvenile stage of *Citrus* plants when regenerated from juvenile explants (epicotyls, cotyledon or calli), which delays the observation of the resulting phenotype. Alternatives include the use of explants from adult tissues, which sometimes may be recalcitrant to regeneration or transformation, or of early-flowering genotypes. However, there is no report about the use of early-flowering sweet orange mutants for functional genomic studies.

**Results:**

Here, we propose a sweet orange spontaneous early-flowering mutant, named ‘x11’, as a platform for *Citrus* functional genomic studies, particularly for genes associated with flower or fruit traits. We report a procedure for efficient regeneration and transformation using epicotyl segment explants of ‘x11’ and *Agrobacterium tumefaciens* as a proof-of-concept. The average transformation efficiency was 18.6%, but reached 29.6% in the best protocol tested. Among 270 positive shoots, five were *in vitro* micrografted and acclimatized, followed by evaluation of transgene expression by quantitative amplification of reversed transcripts (RT-qPCR) and determination of the number of copies inserted. Four of these plants, containing from one to four copies of the transgene, exhibited the first flowers within three months after *ex vitro* establishment, and the other, two months later, regardless of the period of the year. Flowers of transgenic plants displayed fertile pollen and gynoecium, with self-pollination inducing fruit development with seeds. Histochemical staining for β-glucuronidase activity using stem segments, flowers and fruits from 5 to 7 month-old acclimatized transgenic plants confirmed the constitutive transgene expression in these organs.

**Conclusion:**

The ‘x11’ sweet orange is suitable for functional genomics studies with a satisfactory transformation rate, and it can be considered a good model for functional genomic studies in commercial sweet oranges, for traits related to flower and fruit.

**Electronic supplementary material:**

The online version of this article (doi:10.1186/1756-0500-7-511) contains supplementary material, which is available to authorized users.

## Background

*Citrus* species are perennial plants with a long juvenile period, which represents a barrier for conventional breeding by controlled hybridization [1]. The juvenile stage of *Citrus* plants propagated by seeds can last from 5 to 22 years, depending on the species and genotype [2]. Consequently, genetic transformation has been considered an important breeding alternative for *Citrus* as a mean of introducing desirable traits in elite cultivars without affecting a highly favorable genotypic combination, by avoiding recombination during meiosis [3].

There are many reports about gene introduction in several *Citrus* species, mostly involving resistance to biotic [4–7] or abiotic stresses [8, 9]. However, there have been few reports about traits associated with reproductive organs, mostly with fruits, which included attempts to improve fruit skin or pulp [10–12] or the use of antisense of *1-aminocyclopropane-1-carboxylate synthase* gene to transform ‘Carrizo’ citrange, sweet orange and *Poncirus trifoliata* (L.) Raf. to repress the increase of 1-aminocyclopropane-1-carboxylate (ACC) content following chilling treatment of fruits [13]. Regarding flowers, there is a report about transformation of juvenile *Citrus* seedlings to constitutively express the arabidopsis *Leafy* (*lfy*) or *Apetala 1* (*ap1*) genes to promote flower initiation and fruiting as early as the first year after planting [1]. A major reason for the limited number of reports might derive from the extensive juvenile stage of *Citrus* plants when regenerated from juvenile explants, which delays the observation of the resulting phenotype.

Adult-derived explants can be used as an alternative to avoid the extensive juvenile stage of *Citrus* [6, 14–17]. However, the use of mature explants is limited as they are usually more recalcitrant to infection and to genetic transformation by *Agrobacterium tumefaciens*, with low transformation efficiency [12] and the possibility of reduced rooting capacity of the regenerated plantlets [18].

As a consequence of the increasing availability of genomic information, there is an urgent need to facilitate the determination of *Citrus* gene function. *Citrus* functional genetics have been predominantly conducted in model systems, such as tomato and Arabidopsis [19, 20]. Despite the importance and speediness by using these model systems, there are critical differences in species development, gene family structure or individual response for each genotype, which can result in contrasting genetic information [21].

One possible alternative to investigate gene functions in *Citrus* for the observation of flower and fruit phenotypes in a shorter time can be the use of early flowering genotypes, such as ‘Lima West Indian’ [*C. aurantifolia* (Christm.) Swingle] [22]; ‘Kumquat’ (*Fortunella crassifolia* Swingle) [23]; or the spontaneous *Poncirus trifoliata* (L.) Raf. mutant [24], which were all evaluated for genetic transformation [12, 25]. All these species/genotypes offer the potential to be used in functional genomic studies [12], but they are less attractive than sweet orange genotypes [*C. sinensis* (L.) Osbeck], because of their limited commercial interest.

The spontaneous mutant ‘x11’ was selected from trees of the sweet orange ‘Tobias’, an early-flowering cultivar with polyembryonic seeds [26], grown at the *Citrus* germplasm repository of the ‘Centro de Citricultura Sylvio Moreira’, Cordeirópolis, São Paulo, Brazil by Dr. Rodrigo R. Latado in 2006. The mutant sport was subsequently budded to select for a solid (non-chimerical) mutant plant. ‘x11’ differs from ‘Tobias’ as a more compact plant, with fruits with higher seed number (approximately 6.0) (R.R. Latado, unpublished observations). Seedlings of ‘x11’ bloom repeatedly in all seasons, without the requirement of environmental stimuli, except for pruning, which stimulates the production of new shoots (18-24 cm long; 9-12 leaves), usually with a terminal flower, 30-40 days later. Male and female organs are viable (ranging from 53 to 92% fertility, according with the season), and pollen germination rate varies between 25 to 55%, resulting in an easy fruit set after pollination (R.R. Latado, unpublished observations). Despite the fact that ‘x11’ seedlings produce complete and fertile flowers, abortion tends to occur when the plants are still small. However, if juvenile buds are grafted onto large or adult plants, the rate of fruit set reaches a normal level. The average yield of ‘x11’ plants still needs to be evaluated upon field conditions, but there is an expectation to be similar to the one observed for ‘Tobias’, of approximately 61 kg plant^-1^ year^-1^ [26]. These attributes make ‘x11’ an attractive functional genomic model to investigate gene functions associated with flower and fruit development and traits in a shorter period of time (one to two years) in sweet orange.

Despite the significant progress in establishing genetic transformation protocols by *A. tumefaciens*, some *Citrus* genotypes are still recalcitrant, with low transformation efficiency [10]. Since transformation rate is genotype-specific, there is a requirement to optimize conditions to produce transgenic for each genotype [27]. Here, we described the genetic transformation of the early-flowering sweet orange ‘x11’ using *A. tumefaciens* with a reporter gene *β-glucuronidase* (*uidA*) driven by the 35S cauliflower mosaic virus (CaMV) promoter as a proof-of-concept for adopting this genotype as a platform for functional *Citrus* genomic studies of flower and fruit-related gene function analyses in *Citrus*, particularly sweet orange.

## Results and Discussion

### Genetic transformation of ‘x11’ sweet orange

Several experiments were previously conducted to optimize the conditions of genetic transformation of ‘x11’ epicotyl explants, including the determination of 6-benzylaminopurine (BAP) concentration on the regeneration media; kanamycin concentration for selection of transgenic events; and inoculation and co-cultivation conditions (Additional file 1 Table S1). The best transformation and regeneration conditions for epicotyl segments of ‘x11’ sweet orange tested, resulted in regeneration efficiency of shoots ranging between 1.0 and 3.8 shoots per explant in experiments without co-cultivation with *A. tumefaciens* (control), or 0.2 to 0.5 shoot per explant, when explants were co-cultivated in bacterial solution. A total of 1,447 explants were used in transformation experiments, resulting in 475 regenerating shoots, from which 270 were positive for GUS staining. GUS-positive shoots represented approximately 57% of the total analyzed shoots, with an average transformation efficiency of 18.6%.

Transformation efficiency of *Citrus* is genotype-dependent, and rates reported have ranged from 2% in ‘Ridge pineapple’ sweet orange [28] to 87.7% in *P. trifoliata* [29]. Transformation efficiency of the protocol defined here for ‘x11’ sweet orange reached in certain experiments up to 29.8% (Supplementary Table 1), similar to rates reported for other sweet oranges, such as ‘Valencia’ (23.8%) [30] or ‘Hamlin’ (25%) [27]; ‘Carrizo’ citrange (20.6%) [31]; or the early-flowering *P. trifoliata* mutant (20.7%) [12], but superior to those described for ‘Mexican’ lime (8%) [27] and sour orange (2.4%) [28].

**Table 1:** **Estimated and assumed copy number of the** ***nptII*** **transgene of five ‘x11’ transgenic plants**

Plants	Estimated copy number	Assumed copy number
Control (Non Transformed)	0.0	0
#1	1.0	1
#2	4.4	4
#3	0.2	1
#4	1.2	1
#5	2.2	2

### Characterization of transgenic shoots and plants

**Figure 1:**
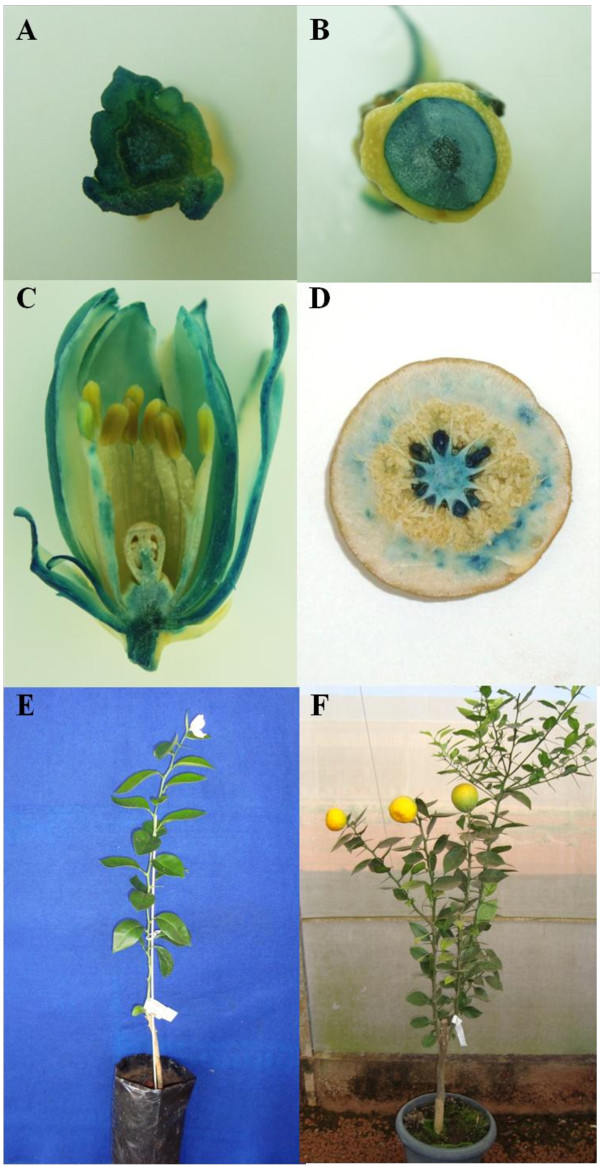
**Tissues of the ‘x11’ sweet orange plant #1 transformed with pCAMBIA2301 expressing** ***uidA*** **gene. A)** cross-section of stem segment; **B)** floral pedicel; **C)** flower after anthesis; **D )** middle section of the fruit; **E)** transgenic ‘x11’ sweet orange plant event #1 at the flowering stage, three months after acclimatization; **F)** transgenic ‘x11’ sweet orange plant with mature fruits, approximately 14 months after acclimatization.

Among the 270 GUS-positive shoots, five were *in vitro* micrografted onto ‘Carrizo’ citrange seedlings and then, acclimatized to greenhouse conditions. Four transgenic plants exhibited the first flowers within three months after establishment *ex vitro* (Figure 1), and the other, two months later (five months after acclimatization), regardless of the time of year. All plants presented the same phenotype, with a terminal flower in almost all developed shoots. Flowers displayed fertile pollen (not shown) and gynoecium, with self-pollination inducing fruit development with seeds.

Histochemical staining for *uidA* (GUS) activity using stem segments, flowers and fruits from 5 to 7 month-old acclimatized transgenic plants confirmed the constitutive expression of *uidA* gene in these organs (Figure 1). PCR analyses of the five putative transgenic (GUS-positive) plants indicated the presence of a 203 bp fragment, equivalent to the expected amplicon of the *neomycin phosphotransferase* (*nptII*) gene (not shown). When these plants were analyzed for the number of copies of transgenes inserted, the evaluated amplification efficiency of the primers for the target gene (*nptII*) and for the endogenous reference gene (*lipid transfer protein* - *ltp*) was around 100%. The virtual calibrator (r_1_ coefficient) was calculated for *nptII* as described by Mason et al. [32], and in this experiment, the r_1_ value was estimated to be 0.8. Thus, it was estimated that among the five putative transgenic plants analyzed, the events #1, #3 and #4 presented a single copy of the transgene *nptII*, while the plant #5 contained two copies and the plant #2 showed four copies (Table 1). The copy number of transgene in event #3 was estimated to be 0.2 using the method described by Mason et al. [32]. However, it was demonstrated by GUS histochemical staining, PCR amplification and *nptII* gene expression analyses that this plant is transgenic. Therefore, it was assumed that one single copy of the the *nptII* transgene was inserted.

This type of uncertainty in estimating the number of copies of transgenes has been observed in several reports [32–35]. This may occur in some cases due to fact that the qPCR reaction cannot detect rearrangements of T-DNA during insertion into the host chromosome or because the possible occurrence of partial loss of the transgene in the expression cassette. Nevertheless, this method can be considered as simple, fast, efficient and with high sensitivity in comparison with other methods, such as Southern blot and, therefore, is considered reliable to estimate the transgene copy number [32].

**Figure 2:**
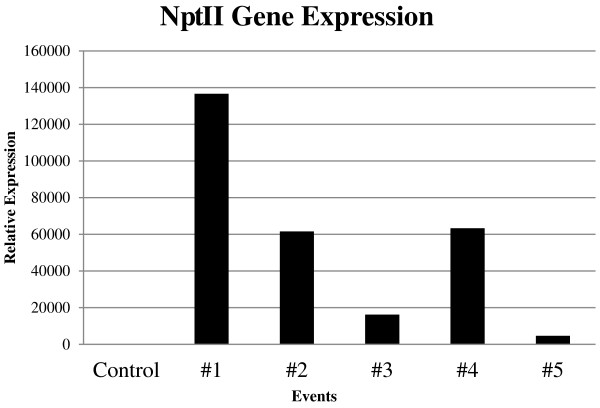
**Relative expression of the** ***nptII*** **gene.** Relative expression of the *nptII* gene in control and in transgenic plants #1, #2, #3, #4 and #5, transformed with pCAMBIA2301, in relation to the gene encoding *Eukaryotic Translation Initiation Factor* 5A (*IF5A*) used as a reference gene.

The level of transgene expression varied between the events evaluated, with the highest number of transcripts accumulated observed in event #1 (~135,000x more than the control plant; Figure 2), which contained a single copy of the transgene, while the smallest number of transcripts was detected for event #5 (Figure 2), with estimated two copies of the transgene. Previous studies have indicated that plants with larger number of transgene copies resulted in a lower level of transgene expression, unstable expression or even gene silencing [36, 37]. On the other hand, the insertion of only one or two copies tends to result in higher levels of expression [38, 39].

### Prospects for sweet orange functional genomics

With the completion of the genome sequences from important *Citrus* species [ [40], http://www.citrusgenomedb.org/], together with the availability of vast amount of expressed sequences [41, 42] and protein data [43, 44], there is an urgent requirement for establishing a functional genomic platform to uncover several gene functions, involved in structural, signaling and regulatory pathways in sweet orange fruits, the economic focus of *Citrus* production. Many plant species have been used as an ortologous model system, such as arabidopsis and tomato, to investigate *Citrus* gene functions [19, 45]. However, these model systems may differ for specific gene function, or regulation and signaling. The evaluation of specific promoters is also hampered in ortologous model systems.

Our results indicated that the sweet orange mutant ‘x11’ is suitable for functional genomic studies. We showed that ‘x11’ is able to blossom and produce fruits around 5 months after hardening upon greenhouse conditions, expressing the transgene. Other species have been proposed to be used in functional genomics studies, including the early-fruiting *P. trifoliata* [12, 25], or the short juvenile phase *C. aurantifolia* [22] and kumquat (*Fortunella* sp.) [23]; however, none of these species exhibit the large commercial interest as the sweet orange ‘x11’, the short juvenile stage, nor comparable transformation efficiency described here. Seeds from ‘x11’ can be provided in limited amounts upon request for research purposes only.

## Conclusion

The results demonstrated the concept that the early-flowering sweet orange ‘x11’ mutant appears as a suitable genotype for functional genomic studies of target genes involved in the processes of flowering and fruiting, enabling the quick evaluation of resulting phenotypes.

## Methods

### Plant material and explant source

Seeds from the early-flowering sweet orange mutant ‘x11’ were obtained from field-grown plants. In a laminar flow-hood, seed coat was removed and embryos were superficially disinfected in 50% commercial solution of sodium hypochlorite (final concentration of 1-1.5% active chlorine). Embryos were then germinated in test tubes containing 10 mL semi-solid MS media supplemented with 7 g L^-1^ agar. Tubes were maintained in the dark for 30 days at a 25 ± 1°C for epicotyl elongation, followed by cultivation under light (50 μmol m^-2^ s^-1^ at 16 h photoperiod) for another 15 days. One-cm epicotyl segments were then cut and used as explants.

### Transformation vector

*A. tumefaciens* strain EHA105 [46] containing pCAMBIA2301 [http://www.cambia.org.au] plasmid was used for transformation. The plasmid contained the plant selection gene *nptII* and the reporter gene *uidA* under the control of the CaMV35S promoter and the *nopaline synthase* (*nos*) gene terminator.

### Genetic transformation of ‘x11’ explants

To improve the efficiency of epicotyl segment regeneration and transformation of ‘x11’ explants some parameters were evaluated in preliminary experiments, such as: BAP 6-benzylaminopurine concentration in regeneration medium; kanamycin concentration used for transgenic selection; inoculation time of *Agrobacterium*; co-cultivation temperature and days of co-cultivation (Additional file 1 Table S1). The efficiencies and quantities of explants evaluated are presented at Supplementary Table 1. In the recommended protocol for genetic transformation experiments, *A. tumefaciens* cells were grown in 20 mL LB media, supplemented with 50 mg L^-1^ kanamycin and 100 mg L^-1^ rifampicin for 16 h at 28°C on an orbital shaker (120 rpm). The bacteria suspension was centrifuged at 5,000 *g* for 15 min, and the pellet was resuspended in T1 media [MS salts and vitamins; 25 g L^-1^ sucrose; 0.5 g L^-1^ malt extract (Sigma; Saint Louis, MO, USA) and 0.1 g L^-1^ myo-inositol] supplemented with 0.5 mg L^-1^ 2,4-dichlorophenoxiacetic acid (2,4-D) and 200 μM acetosyringone at pH 5.4, to OD_600_ = 0.6. Epicotyl segments were excised and exposed to the *Agrobacterium* suspension under agitation at 100 rpm. The period of co-cultivation time was 30 min (co-cultivation time previously tested 10 - 30 min). The excess suspension was dried off using sterile filter paper. Explants were then co-cultivated with *Agrobacterium* on T1 media supplemented with 1.5 mg L^-1^ BAP, 0.01 mg L^-1^ 2,4-D, 100 μM acetosyringone and 7 g L^-1^ agar (pH 5.8) in the dark, at 25 ± 1°C (temperatures of co-cultivation previously evaluated: 22 – 28°C), for three days (period of co-cultivation previously evaluated: one - four days). Then, explants were transferred to fresh T1 media supplemented with 1.5 mg L^-1^ BAP; 100 μM acetosyringone; 7 g L^-1^ agar (pH 5.8); 500 mg L^-1^ cefotaxime and 50 mg L^-1^ kanamycin (kanamycin concentrations previously tested for inhibition of shoot regeneration: from 0 to 150 mg L^-1^). Explants were kept at 25 ± 1°C under 50 μmol m^-2^ s^-1^ and 16 h photoperiod, transferring to fresh media every 15 days until shoot regeneration.

Three experiments of genetic transformation were performed using the same procedures. The number of regenerated shoots (more than 4 mm) per explants was evaluated after 45 days of cultivation and the regeneration efficiency was calculated. All 60-day-old shoots were individually analyzed using histochemical GUS staining [47], and the transformation efficiency was estimated by the ratio number of GUS-positive shoots over the number of inoculated explants, and the percent of GUS-positive shoots.

### Acclimatization and confirmation of transgenic plants

Some GUS-positive shoots transformed with pCAMBIA2301 were *in vitro* micrografted onto ‘Carrizo’ citrange and transferred to liquid T1 media supplemented with 25 g L^-1^ sucrose, at 25 ± 1°C, under 50 μmol m^-2^ s^-1^ and 16 h photoperiod, for 15 days. Plantlets were acclimatized and transferred to 20 L pots with a 1:1 mixture of soil and substrate in the greenhouse. Stem segments, first flowers and fruits of transgenic plants for pCAMBIA2301 were sampled and used for GUS histochemical staining.

Transformation was confirmed by amplification using total genomic DNA, extracted according to Doyle and Doyle [48]. Specific primers for the *nptII* gene (For: CAATAGCAGCCAGTCCCTTC and Rev: AGACAATCGGCTGCTCTGAT) were developed using Primer3 [49], with an expected amplicon of 203 bp. The amplification reaction was conducted on a GeneAmp 9700 thermocycler (Applied Biosystems; Foster City, CA, USA) in a final volume of 25 μL with 25 ng DNA in *Taq* buffer containing (NH_4_)_2_SO_4_ [75 mM Tris-HCl, pH 8.8; 20 mM (NH_4_)_2_SO_4_]; 2 mM MgCl_2_; 200 μM of each dNTPs; 0.2 μM of each primer and 1 U *Taq* polymerase (Fermentas; Burlington, Canada). The amplifications started at 95°C for 2 min, followed by 29 cycles of 30 s at 95°C; 30 s at 60°C; 40 s at 72°C, followed by a final extension of 5 min at 72°C. Products were detected by 1% agarose gel electrophoresis.

### Estimation of number of transgene copies inserted and analysis of gene expression by quantitative amplification of reversed transcripts (RT-qPCR)

Five transgenic plants were analyzed by real time PCR method using SYBR Green to determine gene expression and to estimate the number of inserted copies. To estimate the number of transgene copies, primers for the *nptII* transgene (primers above) and for the endogenous *ltp* gene [GenBank AF369931] (ACACCTGACCGCCAAACT and AAGGAATGCTGACT CCACAAG; amplicon size = 115 bp), present as two copies in *C. sinensis* genome [50, 51], were used in amplification reactions with genomic DNA from five transgenic plants plus the respective control plant. The qPCR amplification reactions were performed in triplicate, in a final volume of 10 μL containing 1 μL cDNA 1:10 (v/v) dilution; 0.5 μM of each transcript-specific primers and 5 μL 2X Platinum SYBR-Green RT-qPCR SuperMix-UDG (Invitrogen; Carlsbad, CA, USA). Estimation of copy number of the transgene was conducted as described by Mason et al. [32] and Omar et al. [33]. Standard curves were prepared for the *nptII* transgene and for the endogenous *ltp* gene. These levels were compared with the experimental estimation in control and transgenic samples, and the amount of transgene was divided by the value of the endogenous gene. Then, the r_1_ coefficient (called ‘virtual calibrator’) was calculated for the *nptII* transgene using data from all transgenic and control plants, as described by Mason et al. [32].

For gene expression analysis, total RNA was extracted from five putative transgenic plants and from one non-transgenic plant according with the protocol described by Tao et al. [52]. The target transgene was *nptII* (primers above) and the reference gene was the *Eukaryotic Translation Initiation Factor 5A* (*IF5A*) [TIGR: TC17010] (ACTGAAACCGGAAACACCAA and TTTCCTTCAGCAAACCCATC; amplicon size 89 pb). cDNA synthesis was conducted as described by Pinheiro et al. [53] and the RT-qPCR reactions were performed as described above in the experiment for analysis of *nptII* transgene expression. Amplifications were performed in a RotorGene 3000 thermocycler (Corbett Life Science; Sidney, Australia) in triplicates, with initial incubation at 50°C for 2 min and 95°C for 2 min, followed by 40 cycles of 95°C for 15 s; 60°C for 15 s, and 72°C for 20 s, with fluorescence detection at the end of the extension cycles. After the final cycle, melting curves for each amplicon were determined between 72 and 95°C.

## Electronic supplementary material

Additional file 1: Table S1: Summary of optimizing the conditions of genetic transformation of ‘x11’ epicotyl explants. Evaluation of number of explants and explants with shoots; number of regenerated shoots, efficiency of shoot regeneration; number of GUS-positive shoots; efficiency of transformation and percent of GUS-positive shoots, of ‘x11’ sweet orange, obtained in three independent experiments. (DOCX 38 KB)
