# Supplementary material for: Early-flowering sweet orange mutant ‘x11’ as a model for functional genomic studies of Citrus
Source: BMC Res Notes. 2014 Aug 10;7:511. doi: 10.1186/1756-0500-7-511 (PMC4267115; doi:10.1186/1756-0500-7-511)
Supplement: Supplementary file 1 — Additional file 1: Table S1: Summary of optimizing the conditions of genetic transformation of ‘x11’ epicotyl explants. Evaluation of number of explants and explants with shoots; number of regenerated shoots, efficiency of shoot regeneration; number of GUS-positive shoots; efficiency of transformation and percent of GUS-positive shoots, of ‘x11’ sweet orange, obtained in three independent experiments. (DOCX 38 KB) [file 13104_2014_3041_MOESM1_ESM.docx]

Supplementary Table 1. Summary of optimizing the conditions of genetic transformation of ‘x11’ epicotyl explants. Evaluation of number of explants and explants with shoots; number of regenerated shoots, efficiency of shoot regeneration; number of GUS-positive shoots; efficiency of transformation and percent of GUS-positive shoots, of ‘x11’ sweet orange, obtained in three independent experiments ^w^.

| **Evaluated Factor** | | **Treatments** | | **No. of explants cultivated** | | **No. of explants with shoots** | | **No. of explants with regenerated shoots** | | **Efficiency of shoot regeneration ^x^** | | **No. of GUS positive shoots (% ^y^)** | | **Efficiency of transformation ^z^** |
| --- | --- | --- | --- | --- | --- | --- | --- | --- | --- | --- | --- | --- | --- | --- |
|  | 0 mg L^-1^ | | 141 | | 133 | | 146 | | 1.04 c | | - | | - | |
| **BA concentration** | 0.5 mg L^-1^ | | 176 | | 171 | | 428 | | 2.40 b | | - | | - | |
|  | 1.5 mg L^-1^ | | 163 | | 158 | | 633 | | 3.84 a | | - | | - | |
|  | 3.0 mg L^-1^ | | 127 | | 124 | | 493 | | 3.82 a | | - | | - | |
|  | 0 mg L^-1^ | | 100 | | 96 | | 189 | | 1.89 | | - | | - | |
| **Kanamycin** | 50 mg L^-1^ | | 100 | | 0 | | 0 | | 0 | | - | | - | |
| **concentration** | 100 mg L^-1^ | | 100 | | 0 | | 0 | | 0 | | - | | - | |
|  | 150 mg L^-1^ | | 100 | | 0 | | 0 | | 0 | | - | | - | |
| **Inoculation** | 10 min | | 189 | | 45 | | 92 | | 0.49 ns | | 44 (47.8%) | | 23.3% ns | |
| **time of** | 20 min | | 171 | | 38 | | 73 | | 0.43 ns | | 36 (49.3%) | | 21.1% ns | |
| ***Agrobacterium*** | 30 min | | 99 | | 19 | | 37 | | 0.37 ns | | 24 (64.9%) | | 24.2% ns | |
|  | 22 °C | | 165 | | 26 | | 41 | | 0.25 ns | | 27 (65.9%) | | 16.4% ns | |
| **Co-cultivation** | 25 °C | | 172 | | 32 | | 54 | | 0.31 ns | | 42 (77.8%) | | 24.4% ns | |
| **temperature** | 28 °C | | 168 | | 22 | | 32 | | 0.19 ns | | 15 (46.9%) | | 8.9% ns | |
|  | 1 day | | 134 | | 15 | | 22 | | 0.16 b | | 16 (72.7%) | | 11.9% b | |
| **Days of** | 2 days | | 118 | | 22 | | 49 | | 0.42 a | | 35 (71.4%) | | 29.7% a | |
| **co-cultivation** | 3 days | | 108 | | 13 | | 27 | | 0.25 ab | | 11 (40.7%) | | 10.2% b | |
|  | 4 days | | 123 | | 17 | | 48 | | 0.39 ab | | 20 (41.7%) | | 16.3% ab | |
| **Total or average of transformation experiments** |  | | 1447 | | 249 | | 475 | | 0.33 | | 270 (56.8%) | | 18.6% | |

**^w^** The experimental design using in these experiments was completely randomized, and the regeneration and transformation efficiency data was transformed (√
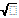
x + 0.5) before analysis of variance (ANOVA), considering as factors: treatment, experiment and interaction, using the GLM procedure of SAS version 6.11;

**^x^** Number of regenerated shoots relative to inoculated explants;

**^y^** Calculated as the ratio between the number of GUS positive shoots and the total number of inoculated explants;

^z^ Calculated as the ratio between the number of GUS positive shoots and the total number of regenerated shoots.
